# Hospital Bed Occupancy and HIV/AIDS in three Major Public Hospitals of Addis Ababa, Ethiopia

**Published:** 2010-09

**Authors:** Melesse Tamiru, Jemal Haidar

**Affiliations:** 1 *School of Public Health, Addis Ababa University, Ethiopia;*; 2 *School of Public Health, Addis Ababa University, Ethiopia*

**Keywords:** HIV/AIDS, hospitals, bed occupancy, Addis Ababa

## Abstract

**Background:**

In countries like Ethiopia where the spread of HIV infection is extensive, health services are faced with an increased demand for care. The most obvious reflection of this increased demand is through patient load, longer bed occupancy perhaps to the exclusion of patients with other ailments.

**Objective:**

The purpose of this study was to describe the bed occupancy rate and the average length of stay of HIV/AIDS inpatients of three major public hospitals.

**Methods:**

A Retrospective Cross-sectional study was conducted in three major hospitals of Addis Ababa namely Zewditu Memorial Hospital, Tikure Anbessa Hospital and Saint Paul’s Hospital from February to March 2004.

**Results:**

Of the total 453 sampled inpatients, 293 (65 %) were HIV positives. Over half (55.0%) were Males. The most affected age group was between 24 and 56 years. The majority (85.8%) were from Addis Ababa and over half (57.7%) was married. Housewives constituted about a quarter (26.3%) of all the admitted cases. The most common co-morbidities resulted in admission to the medical wards among the HIV-positive cases were Tuberculosis (73.0%) and jirovicii pneumonia (70.3%), and their occurrence was significantly higher among HIV+ than their counter parts (*p*=0.001). Although numbers of patients admitted in Tikur Anbesa hospital was more than Saint Paul’s and Zewditu Memorial hospitals (ZMH), the proportion of HIV positive cases admitted to ZMH however was higher (49.0%) than Tikur Anbessa (14.0%) and Saint Paul’s hospitals (18.0%). Likewise the number of inpatient days was also higher in ZMH (n=7765) than the other hospitals. The bed occupancy rate was however, higher in ZMH (53.0%) than Tikur Anbessa (12.0%) and Saint Paul’s (12.0%) hospitals.

**Conclusion:**

One of the most obvious consequences of HIV/AIDS patients are the increased occupancy of hospitals beds suggesting that only 81.1 % of the beds are for all other afflictions in the hospitals. It appears that there is a lot of concern that patients with HIV are competing with the non-HIV infected patients in a resource limited areas. Home based care with community involvement and greater use made of existing community resources might be a response to the limitations of curative hospital-based care and treatment needs of many HIV/AIDS patients.

## INTRODUCTION

In all heavily affected countries the AIDS epidemic is adding additional pressure on the health sector (1). As the HIV prevalence of a country rises, the strain placed on its hospitals is likely to increase. In sub-Saharan Africa, people with HIV-related diseases occupy more than half of all hospital beds (2). According to the Government-funded research conducted in South Africa, HIV positive patients stay in hospital four times longer than other patients on average (3).

AIDS affects health care sector through both supply and demand-sides. As more and more people infected with HIV develop opportunistic infection, this increases the demand for medical care and heavily taxing the over stretched public health services of many developing countries. In the mid 1990s, it was estimated that treatment for people with HIV consumed 66% of public health resources in some African countries like Rwanda and over a quarter of health expenditure in Zimbabwe (4). In recent years, HIV positive patients have occupied half of the beds in the provincial Hospital in Chiang Mai, Thailand, likewise 39% of the beds in Kenyata National Hospital, Kenya and 70% of the beds in the Prince Regent Hospital of Bujumbura, Burundi. A related impact of the epidemic is that patients suffering from other conditions are being crowded out (4).

Also in Cote d’Ivoire, Zambia, and Zimbabwe HIV-infected patients occupy 50 to 80 percent of all available beds in urban hospitals implying that the services provided meet only a fraction of the general needs suggesting availability of hospital bed has always been a problem in developing countries (5, 6). The availability of beds is perhaps the most important single factor in determination of the hospital utilization in country (6). In India, shortage of hospital beds has also been mentioned a huge problem with an average bed population ratio of 6.8/10,000 (7).

In Ethiopia, previous national studies dealt with the prevalence of HIV infection in the country and data on bed occupancy rate (BOR) and average length of stay (ALOS) of HIV infected patients however are non existent and appears a neglected area. This necessitated the present study to explore the affirmation gaps of BOR and AOLS because they are important for establishing a framework for prioritizing health services in the country. In addition dealing with BOL and ALOS will help to lay a ground to design an efficient plan and match our desires and ideas more realistically with the present resource situation (8).

Given the health needs of population and the HIV/AIDS epidemic together with the growth of population are placing unprecedented demands on public hospitals will make this study extremely crucial to assess these institutions with regard to their efficiency in allocating scarce resources (9-12). In this regard, Bed occupancy and performance assessment would presumably provide managers with the information they need for evaluation and monitoring of the hospital’s current status and activities (9). The purpose of this study was therefore to shade light on such neglected issue and yet important for the hospital authorities and policies makers for future endeavor.

## METHODS

### Study Area

The study was done in Addis Ababa, the major urban centre, the capital city of the country. The population of Addis Ababa as of 2008 was 3,384,569 with a population growth rate of nearly 2.9% per year (10). More than 50 percent of the health facilities of the country are located in Addis Ababa showing that the situation is better than in the rest parts of the country (11). Much of the population growth in the city still stems from migration from the countryside and smaller peri-urban areas. Because of these, unemployment is high and incomes are low and substandard housing conditions with poor sanitation are becoming a challenge in some part of the city. Presence of large numbers of commercial sex workers in the city aggravates the spread of HIV and other sexually transmitted diseases (12). According to a recent report, 60% of the households in the city earn less than Birr 300 per month (13). There were 5 public and 25 private hospitals in the city. Of the total 5 public hospitals, 3 of them who had large clients were purposively selected and included in the present assessment of bed occupancy study. The names of hospitals were Zewditue Memorial, Tikur-Anbessa and Saint Paul’s. These hospitals are managed by Addis Ababa Health Bureau, Addis Ababa University and Federal Ministry of Health respectively. All the 3 hospitals served over 120,000 patients yearly and involved in the fight against the spread of the virus. The hospitals also provide a wide range of services such as Voluntary Counselling and Testing (VCT), prevention of mother-to-child transmission, anti-retroviral therapy, treatment of opportunistic infections and other curative services. Only medical wards were considered and included for our present assessment, since HIV/ AIDS patients are mainly managed in the medical departments.

### Study Design

This is a facility based retrospective cross sectional study.

### Study Population

All HIV positive patients whose age was ≥14 years admitted in medical wards of the three hospitals from July 2002 to June 2003 were included.

### Sample Size

The sample size was estimated by assuming 50% prevalence of BOR (since there is no prevalence data) with 5% margin of error, 95% confidence level and 50% an allowance for missing information. A total of 460 cases were required for this assessment. The numbers of in-patient medical cards were drawn from each hospital based on their total number of HIV tested patient sizes using proportional probability sampling (PPS) as shown below.

### Data Collection and Management

Check list was developed to collect data on socio-demographic characteristics, hospital stay of patients and co-morbidities. Medical records of admitted patients were then reviewed and screened for patients’ sero-positivity. Data were collected by six trained health workers (councillors) serving as data collectors. Prior to data collection, the prepared cheek list was pre-tested in Tikuranbessa hospital on few medical records of patients from the archive by the principal investigator (PI). After few adjustments and refinements were made, the final version of the cheek list was developed in English. Data collectors were then trained and standardized their method of data collection during the three days extensive training by the PI. Data were then collected from the archives of each hospital using the structured cheek list under close supervision of the PI. The manually edited data were then entered in to a computer and analysed using SPSS software version 15. Frequencies, percentages and mean were used for descriptive analysis. Pearson’s Chi-square test was performed to test the existence to significant association of co-morbidities with sero-positivity status of the admitted patients while ANOVA was used to see the association of major co-morbidities with the mean length of stays. A p value of < 0.05 was considered statistically significant. BOR was calculated based on total number of beds (B) and patient days in one year (H) denoted as BO=[H/(365*B)]*100.

### Ethical considerations

This study was approved by Addis Ababa University for its ethical and scientific merit. Medical directors, head of the medical wards of the respective hospitals were informed about the objective and the importance of the study and permitted the study. Anonymous data were collected from the archives by selecting the medical wards’ records by trained health workers recruited for this study and involved in the management of the HIV/AIDS patients of respective hospitals.

### Operational Definitions

**Average length of stay.** the average number of days patients stayed in each ward and in the hospital. To calculate this parameter, in-bed days in each ward were divided by the output of wards. The output was the sum of the discharged, deceased and transferred patients.

**In-bed days.** total number of active beds in each ward, which were occupied by patients, calculated per day, month or year.

**Bed occupancy rate.** percentage of beds in each ward occupied by patients during one year.

## RESULTS

Table 1 describes the socio-demographic characteristics of the patients by their HIV status. Out of the total 453 sampled patients, 293 (64.7%) were HIV positive while the rest 160 (34.3%) were HIV negative. Of the 293 HIV positive patients, 55% were males. The most affected age group was ranging from 24 to 56 years. The majority (85.8%) of HIV positive patients were residents from Addis Ababa. Over half (57.7%) of the HIV positive patients was married. The proportion of HIV positive patients, who were housewives, civil servants, drivers and daily labourers, was 26.3%, 22.8%, 14.6% and 13.4% respectively.

**Table 1:** Socio-demographic Characteristics of Patients by HIV Status in the three major hospitals (Tikur Anbessa, Saint Paul’s, Zewditu Memorial), Addis Ababa, 2004

Variables	Total	HIV positive n (%)	HIV negative n (%)

Sex			
Male	243	162 (55)	81 (51)
Female	210	131 (45)	79 (49)
Age			
14-23	57	21 (7.2)	36 (22.8)
24-34	169	115 (39.5)	54 (34.2)
35-45	120	86 (29.6)	34 (21.5)
46-56	75	53 (18.2)	22 (13.9)
57-67	19	11 (3.8)	8 (5.1)
>67	13	7 (1.5)	6 (2.5)
Place of Residencea			
Addis Ababa	328	225 (85.8)	103 (74.8)
Out of Addis	75	39 (14.2)	36 (25.2)
Marital Statusa			
Married	170	112 (57.7)	58 (52.2)
Single	97	51 (26.2)	56 (41.4)
Divorced	20	16 (8.2)	4 (3.6)
Widowed	18	15 (7.7)	3 (2.7)
Occupation^a^			
Civil Servant	69	45 (26.3)	24 (30.3)
Housewife	41	39 (22.8)	2 (2.5)
Daily Labourer	34	23 (13.4)	11 (13.9)
Student	28	11 (6.4)	17 (21.5)
Driver	27	25 (14.6)	2 (2.5)
Unemployed	17	11 (6.4)	6 (7.5)
Merchant	13	13 (7.6)	0
NGO	6	4 (2.3)	2 (2.5)

a Total sample size doesn’t add up to 453 because of missing information from patient cards.

Table 2 displays the types of clinical conditions required admissions disaggregated by HIV status of the patients. The most common co-morbidities resulted in admission among the HIV-positive cases at admission were Tuberculosis (40.6%) followed by jirovicii pneumonia (9.9%) toxoplasmosis (6.6%), chronic diarrhoea (4.4%) and oral thrush (1.5%). The proportion of patients suffered from peptic ulcer diseases (4.4%), acute febrile illness (1.9%) and septic shock (2.4%) cases were more in the HIV negatives than their counterparts. The occurrence of TB and jirovicii pneumonia was significantly higher among the HIV positive than HIV negatives admitted patients.

**Table 2:** Diagnosed morbidities during admission in HIV Positive and Negative patients of three major hospitals (Tikur Anbessa, Saint Paul’s, Zewditu Memorial), Addis Ababa, 2004

Type of comorbidities	HIV positive n (%)	HIV negative n (%)	Total	X^2^	*p*

Tuberculosis (TB)	184 (40.6)^a^	68 (15.0)	252 (55.6)	16.4	0.001
Toxoplasmosis	30 (6.6)	21 (4.6)	51(11.2)	1.63	0.2
Jirovicii pneumonia	45 (9.9)^a^	19 (4.1)	64(14.0)	0.77	0.3
Chronic diarrhoea	12 (4.4)	8 (1.8)	20(6.2)	0.004	0.8
Oral Thrushes	7 (1.5)	4 (0.9)	11(2.4)	0.06	0.8.
Peptic ulcer disease	8 (1.8)	20 (4.4)	28(6.2)	15.39	0.001
Septic shock	4 (0.9)	11 (2.4)	15(3.3)	8.17	0.001
Acute febrile illness	3 (0.6)	9 (1.9)	12(2.5)	6.81	0.001

a *p*=0.001.

**Figure 1:**
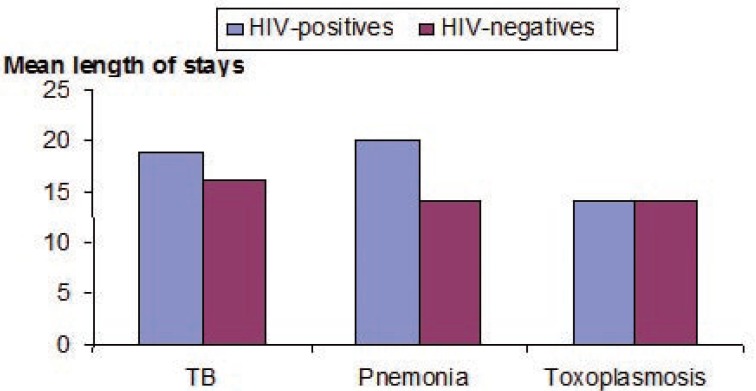
Mean length of stays by Co-morbidities in three major hospitals^a^, Addsi Ababa, 2004. ^a^Tikur Anbessa, Saint Paul’s, Zewditu Memorial; ANOVA=0.49; *p*=0.78.

Figure 1 displays the mean length of stays for the major co-morbidities by HIV status. Although the occurrence of TB (19 days) and Jirovicii pneumonia (20 days) was higher in the HIV-positives than HIV negatives, the difference noted however, was not statistically significant (*p*=0.7).

Table 3 denotes the total number of HIV positive patients’ admitted to medical ward of the three hospitals. Overall, 4063 patients were admitted. Of theses, 810 (21.4%) were HIV seropositives. When the admission was disaggregated by hospitals, Tilkur Anbesa hospital admitted a large number (n=2066) of patients than Saint Paul’s (n=1301) and Zewditue Memorial (ZMH) hospital (n=696). However, the number of HIV-positives patients admitted was more in ZMH (n=696) than Tikur Anbessa (n=290) and Saint Paul’s (n=240) hospitals. Likewise the proportion of HIV-positive patients admitted to ZMH was higher (49.0%) than Tikur Anbessa (14.0%) and Saint Paul’s hospitals (18.0%).

**Table 3:** Number of total admissions and HIV positives patients of the three major hospitals, Addis Ababa, 2004

Names of the hospitals	Total number of admissions of patients	Total no of HIV Positive patients	Proportion of HIV positive patients

Tikur Anbessa Hospital	2066	290	14.0
Saint Paul’s Hospital	1301	240	18.0
Zewditu Memorial Hospital	696	340	49.0
Total	4063	870	21.4

Table 4 shows the overall number of inpatients days, number of beds available and bed occupancy of soro-positive patients. The total number of inpatient days was 16,520 days with an overall bed occupancy rate of 18.9%. The number of inpatient days was higher in ZMH (n=7765) than the other hospitals. The number of beds were more in Tikur Anbessa (n=123) than Saint Paul’s (n=77) and ZMH (n=40) hospitals. The bed occupancy rate was however higher in ZMH (53.0%) than Tikur Anbessa (12.0%) and Saint Paul’s hospitals.

**Table 4:** Percentage of beds occupied on average by HIV/AIDS patients of the three major hospitals (Tikur Anbessa, Saint Paul’s, Zewditu Memorial), Addis Ababa, 2004

Characters	Total	Tikur Anbessa hospital	Saint. Paul’s hospital	Zewditu Memorial hospital

Inpatient days of HIV cases (H)	16520	5375	3380	7765
No of Beds available (B)	240	123	77	40
Bed occupancy rate (BO)	18.9	12	12	53

BO = [H / (365*B)]*100.

## DISCUSSION

In most developing countries, hospitals are constrained with high and still increasing rates of bed occupancy by chronic illnesses (14). In most of developing countries hospitals including our studied hospitals, the information that can be extracted from routine statistics are limited and the most commonly available data are date of admission, date of discharge (average length of hospital stay), discharge diagnosis and no information on bed occupancy rates (personal communication with the attending health workers from the medical ward units). Comparisons of admitted cases however, disaggregated by type of morbidities are useful indicative about the existence of problems, but require refined analysis for seeking solutions towards assessment of the use of hospital beds. Cognisant of this fact, we studied the overall medical ward admission of three major hospitals in the capital city of the country. The major interest reflected in our study was to see how the beds were being used and estimate the bed occupancy rates and AOLS including whether any of the patients admitted to the medical wards could have been treated elsewhere or whether they were in hospital primarily for non medical reason, we restricted our discussion on BOR and related information.

According to this study, although the overall share of beds occupied by HIV seropositives is some what higher than most African countries, the actual BOR was markedly lower (18.9%). When the BOR compared with the overall bed occupancy rate of some other African countries like Kinshasa (53%), Kampala (53%) Kigali (53%) and Zambia district hospital (47%) (12), the present figure is lower. The lower BOR observed was because those AIDS patients with terminal illness might have chosen to stay at home or it could be the result of pressures to limit HIV/AIDS patient stays in an over crowded hospitals and the result should be interpreted with some caution as the sampled hospitals may not represent the entire hospitals of the country. Even so, the situation raises a lot of concern given that the available resources for health care are shrinking due to various economic problem and population growth and presumably increasing in demands for care made by HIV-infected patients. As a result hospitals are struggling to cope, especially in our country as well as most African countries where there are often too few beds available. Such bed constraint sometimes results in patients being admitted only in the later stages of illness and reducing the chances of recovery of patients suffered from acute illnesses who could have benefited from the medical beds (15).

Of the 293 medical wards inpatients, overall, 40.6% were TB patients with HIV sero-positivity demonstrating that most of the beds were occupied by TB, followed by pneumonia (9.9%) and toxoplasmosis (6.6%) and the difference was statistically significant (*p*=0.001). when the share of each comorbidites are further disaggregated, 72% (182/252) of the TB patients were HIV positive. This finding almost concurs with the Zambian study (14, 15). Similar pattern of co-morbidities admissions were also seen in the paediatric unit of the ZMH (personal communication with attending physician from the paediatric unit).

The mean length of stay of HIV/AIDS patients being 19 days for TB and 20 days for Jirovicii pneumonia appears to be higher than the HIV negative patients however, the differences noted in their length of stays was not significant. Comparable results of length of stays of 20.1 days was also documented in the Zambian district hospital study among the HIV positive cases and the findings were ascribed to the strength of the health service and the availability of a wide range of drugs and other supplies in public hospitals and hence the HIV/AIDS patients might have not been requested to buy the drugs from private market during the study period (14, 15). When the present finding is compared with the Kinshas (17.7 days), Kampala (13.1 days) and Kigali (16.2 days) (15), the average length of stays found in our study (20.1days) appeared to be higher. The absence of distinct differences in length of hospital stays patterns of the two patient groups examined in Kinshas (17.7 days for seropositives and 16.6 days for seronegatives) and Kampala (13.1 days for seropositives and 11.4 days for seronegatives) studies reflected that some therapeutic options, limited and non-specific basic services were provided to both HIV positives and negatives patients. In some case, it could be possible to referred patients back to the health centres for appropriate follow-up in their near-by facilities. Although follow-up of TB patients are usually handled at health centre level and it could be assumed the bed occupancy burden could have been minimized. However, this has not happened probably because of some social elements which we did not capture in this study. The lesson to draw in the present studies as well as the Zambian study is that if resources are made available some of the co-morbidities like TB could be managed at an outpatient levels and freed the beds for other acute illnesses that could benefit from the available medical wards beds of all the hospitals. Health workers should encourage and adhere to the current TB intervention in the country as the trend observed in other countries was quite useful to minimize the burden of bed occupancy in the country.

For example, the beds to population ratio in Addis Ababa are estimated to be 1:725 (16). Similarly, in South Africa, which is experiencing the fastest growth in HIV/AIDS epidemic in the world, patients are turned away from hospitals due to limited beds (17) indicting that managing co-morbidities could be handled on ambulatory basis. Therefore, all hospitals in general and ZMH in particular, where the bed occupancy was two-folds than the other hospital, should strictly adhere to the protocol of TB patient for better hospital beds utilization. Most importantly HIV/AIDS patients are better managed as an outpatient as they are not treated in the same way as the other patients because some health workers are afraid of the contamination (18).

The expected enormous impact on the demand for hospital services from the HIV epidemic has encouraged interest in identifying possible alternatives to inpatient care in order to relive the hospital sector. One of the most prominent suggestions has been home based care for HIV/AIDS patients. In this model the patients stay as much as possible at home and care is carried out by family members. Home based care has often been termed as a cheaper and better solution than hospital care for HIV/AIDS patients in addition it will reduce the number of in patient days in hospitals (18).

## LIMITATIONS

It is important to note that this type of study relies on recorded medical history and thus the accuracy and completeness of medical records is open to some questions and thus further work is required to address the gaps at national level. Although we have tried to present useful information on BOR and ALOS, the data may not reflect the current situation because of new developments towards the management of HIV/AIDS cases.

## CONCLUSION

The HIV epidemic will result in more and more people having health problems which will increase the burden and pressure on hospitals. One of the most obvious consequences of HIV/AIDS patients are the increased occupancy of hospital beds suggesting that only 81.1% of the beds are for all other afflictions in the hospital. It appears that there is a lot of concern that patients with HIV are competing with the non-HIV infected patients in a resource limited areas. Therefore, home based care with higher level of family members or community involvement and greater use made of existing community resources might be a response to the limitations of curative hospital-based care and treatment needs of many HIV/AIDS patients.

## References

[1] UNAIDS (2002). Report on the global AIDS epidemic. Geneva.

[2] UNAIDS (2006). Report on the global AIDS epidemic: The impact of AIDS on people and societies. Geneva.

[3] Inter Press Service News Agency (ISPN) (2006). Health South Africa: a burden that will only become heavier. ISPN, South Africa.

[4] World Bank (1997). Confronting AIDS. Public Priorities in a Global Epidemic.

[5] UNAIDS (2000). Report on the global AIDS epidemic. Geneva.

[6] Llewellyn-Davies R, Macaulay HMC (1995). Hospital Planning and Administration, WHO Monograph series No. 54, Geneva.

[7] Park K (2002). Text Book of Preventive and Social Medicine.

[8] Asadi AA (1998). Principles of calculating needed beds in each area. Teheran, Islamic Republic of Iran, Planning Council in Curative Affairs, Ministry of Health and Medical Education.

[9] Walker D, Mohammed RL (2004). Producing health service efficiently: a review ofmeasurement tools and empirical applications.

[10] Akashi H, Yamada T, Huot E (2004). User fees at a public hospital in Cambodia: effects on hospital performance and provider attitudes. Social Science & Medicine.

[11] Anonymous (2003). Ministry of Health and Social Service Report. Namibia NationalHealth Accounts.

[12] Shadpour K (2006). Health sector reform in Islamic Republic of Iran. Hakim.

[13] CSA (2007). Census, preliminary http://www.csa.gov.et/pdf/Cen2007_prelimineray.pdf.

[14] Central Statistical Agency of Ethiopia (1999). Addis Ababa City Administration of Health Bureau (AACAHB). HIV/AIDS in Addis Ababa, Background, Projections, Impacts and Interventions. AACAHB, Addis Ababa.

[15] Buve A (1997). AIDS and hospital bed occupancy: an overview. Tropical medicine and international health.

[16] CSA (2001). Ethiopia- Demographic and Health Survey. Addis Ababa.

[17] Buve A, Foster S (1995). Carrying out a bed cencus at a district Hospital in Zambia. Health Policy and Planning.

[18] Hansen K, Chapman G, Chitsike I, Kasilo O (2000). The Costs of HIV/AIDS Care at Government Hospitals in Zimbabwe. Health Policy and Planning.

